# Dog bite injuries and armed conflict-related environmental stressors: a nationwide population-based time-series study

**DOI:** 10.1186/s40621-026-00681-6

**Published:** 2026-04-24

**Authors:** Shon Shabat, Lia Schoenfeld, Adi Turjeman, Sigal Fleishman, Dean Ad-El, Asaf Olshinka

**Affiliations:** 1 Department of Plastic Surgery and Burns, Rabin Medical Center - Beilinson Hospital, 39 Jabotinsky St, Petach Tikva, 4941492 Israel; 2 Plastic Surgery and Burn Unit, Schneider Children’s Medical Center, Petach Tikva, Israel; 3 Research Authority, Rabin Medical Center - Beilinson Hospital, Petach Tikva, Israel; 4 Gray Faculty of Medical and Health Sciences, Tel Aviv University, Tel Aviv, Israel

**Keywords:** Dog bite, Armed conflict, Environmental stressors, Injury prevention, Population-based study

## Abstract

**Background:**

Dog bite injuries pose a substantial public health burden worldwide. Environmental and acoustic stressors may contribute to dog behavioral dysregulation. However, there are as yet no population-level studies evaluating whether the incidence of dog bites increases during periods of armed conflict. The present study was conducted in Israel where the population is routinely exposed to an episodic pattern of high-intensity conflicts (escalation) alternating with periods of calm (de-escalation), providing a unique natural experiment to examine the effect of environmental stressors on population-level modifiers of injury risk.

**Methods:**

This nationwide retrospective observational study covered the decade from 2014 to 2025. Healthcare-encounter data were used to capture dog bite-related diagnoses in both hospital and community settings. Exposure to armed conflict-related environmental stress was operationalized using the number of civil defense sirens per month, categorized as none (0), low (< 500), or high (≥ 500) and aggregated by geographic region (North, Center, South). Monthly dog bite counts were modeled using negative binomial regression adjusted for region, seasonality, age group, sex, and socioeconomic status. Secondary outcomes were hospitalization within 7 days and surgical intervention within 30 days, reflecting injury severity.

**Results:**

A total of 63,285 dog bite-related encounters were identified. Compared to months with no sirens, adjusted dog bite incidence increased by 15% during low-exposure months (incidence rate ratio [IRR] 1.15, 95% CI 1.13–1.18) and by 33% during high-exposure months (IRR 1.33, 95% CI 1.28–1.37), demonstrating a graded exposure-response association. High exposure was associated with increased odds of surgical intervention within 30 days (OR 1.09, 95% CI 1.02–1.16; *P* = 0.013).

**Conclusions:**

This study provides the first population-level evidence linking armed conflict-related environmental stressors to increased dog bite incidence, using a quantitative graded exposure measure rather than a binary conflict-period definition. Dog bite prevention and healthcare preparedness should be taken into consideration in civilian injury mitigation strategies during armed conflict.

## Background

Dog bites are a significant and growing public health concern worldwide, with millions of cases reported annually. In the United States, dog bites rank among the leading causes of nonfatal emergency department visits, with approximately 344,000 encounters per year and an incidence of up to 225 per 100,000 population [1]. Dog bite injuries pose a substantial physical, psychological, and economic burden, particularly among pediatric populations [2]. The etiology is multifactorial, involving dog-specific characteristics such as breed, sex, training, and behavioral traits, in addition to human behavior and environmental stressors that may alter dog responses. The stress exposure may be either acute or chronic [3, 4].

Among the potential environmental stressors, several studies have identified loud noises as major contributing factors to anxiety-related and behavior dysregulation and aggression in dogs [5–7]. The most common noises that elicit fear responses are high-decibel fireworks, thunderstorms, sirens, and explosions [7, 8]. Experimental studies examining controlled acoustic environments reported physiological stress responses in dogs, including elevated cortisol levels and increased heart rate, supporting the biological plausibility of noise-induced behavioral changes [8–10].

Besides such external stimuli, dogs are sensitive to their living conditions and the emotional states and behavioral patterns of their owners. Heightened stress, fear, and anxiety among caregivers may contribute to altered animal handling, reduced tolerance, and more unpredictable interactions, potentially lowering the threshold for aggressive dog responses. During the COVID-19 pandemic, multiple studies reported an increase in dog bite injuries, particularly among children, which were attributed to disrupted routines, increased household stress, reduced supervision, and prolonged close contact between people and pets [11, 12]. These observations underscore how sustained environmental and psychosocial stressors can influence human-animal interactions and increase the risk of dog bite injuries.

Beyond identifying individual-level determinants, dog-bite injuries can be studied using an ecological injury-risk model, examining changes in exposure patterns and susceptibility as a function of multiple, interacting factors across different levels of organization within whole environmental systems.

In this context, armed conflict represents a unique population-level stressor capable of modifying the risk of dog bite injury through different, concurrent, pathways. It may simultaneously alter physical environmental conditions (e.g., high-intensity auditory stimuli including civil defense sirens, explosions, gunfire, and noise generated by aircraft, drones, and incoming or outgoing projectiles), human behavior and routines (e.g., time spent at home and supervision patterns), and psychosocial vulnerability (e.g., heightened anxiety and emotional tension). The stressors may occur as discrete acute events or as repeated and prolonged episodes over weeks or months. Accordingly, research conducted in disaster areas revealed significant behavioral changes in companion animals including increased anxiety, heightened reactivity, and unpredictable conduct [13–15].

Taken together, these findings suggest that armed conflict-related environmental stressors may increase both human and dog stress, thereby modifying human-animal interaction dynamics and increasing the likelihood of bite events, potentially with greater injury severity.

Despite the growing recognition of the behavioral effects of environmental stressors on companion animals, empirical data on the relationship between armed conflict-related environmental stress and dog bite incidence remain limited. To our knowledge, there are no published systematic, population-level studies of armed conflict stressors as modifiers of injury risk. Israel’s recurrent exposure to fluctuating intensities of armed conflict over the past decade, characterized by intermittent periods of escalation interspersed with intervals of relative calm, provides a unique natural experiment to examine this issue. Moreover, the nationwide availability of civil defense siren records makes it possible to use air raid alerts as a precise, objective proxy to quantify acute and cumulative environmental stress exposure, overall and across time and geography.

The primary aim of the present study was to evaluate the association between environmental stress related to armed conflict, as reflected by civil defense siren activity, and the incidence of dog bite injuries requiring medical evaluation. Based on prior evidence linking environmental stressors, acoustic stimuli, and altered human-animal interactions to increased dog reactivity, we hypothesized that higher levels of siren activity would be associated with an increased incidence of dog bite injuries. The secondary aim was to assess whether higher exposure levels were associated with greater injury severity, as reflected by the need for hospital admission or operative management.

## Methods

### Study design and data source

A retrospective, nationwide, observational study was conducted, spanning the decade from 2014 to 2025, during which the Israeli population was exposed to a cycle of high-intensity military conflicts and heightened civil defense siren activity (escalation) interspersed with intervals of calm (de-escalation). Healthcare encounter data were extracted at the end of the study from the research data-sharing platform (powered by MDClone) of Clalit Health Services (CHS), Israel’s largest healthcare organization. CHS insures nearly half the national population, with broad geographic coverage. Its electronic health records databases include longitudinal, de-identified information from multiple care sources. For the present study, dog bite cases were identified by ICD-9 diagnostic codes for dog bite. Case ascertainment was not restricted to emergency department encounters; dog bite diagnoses recorded in community-based settings, including primary care, urgent care, and outpatient clinic visits, were captured as well. All individuals of any age with a documented dog bite diagnosis during the study period were included. Given the use of anonymized, population-level administrative data, no exclusion criteria were applied.

### Outcome measures

The primary outcome measure of the study was the monthly incidence of dog bite-related healthcare encounters, assessed at both the national and regional levels and evaluated in relation to monthly exposure to siren activity. The secondary outcome measure was injury severity in relation to level of siren exposure.

Injury severity was assessed using two clinically meaningful endpoints: hospital admission within 7 days of the index dog bite encounter and surgical intervention within 30 days. Relevant surgical interventions were defined as procedures requiring management in the operating room, including wound debridement, operative wound closure, skin grafting, and local, regional, or free-flap reconstruction. Surgical procedures performed within 30 days of the initial dog bite diagnosis were assumed to be related to the index injury. The need for operative intervention and/or hospital admission was used as a proxy for more severe injuries, reflecting greater tissue damage and clinical complexity.

### Exposure assessment

Data on civil defense siren activity were obtained from the Israel Home Front Command. Siren events were recorded by date and geographic region (North, Center, South) but were available for analysis only in aggregated monthly form, and not at finer temporal resolution for linkage with healthcare records. These regions differ in population distribution and in patterns of exposure to civil defense sirens, with historically higher siren activity in conflict-adjacent areas such as the South and, more recently, the North. Stratification by region was therefore performed to account for geographic variability in exposure and population characteristics. Siren activity was categorized into three predefined groups: months with no sirens, months with < 500 sirens (low exposure), and months with ≥ 500 sirens (high exposure). The exposure thresholds were defined a priori based on the structure of the available dataset and were intended to reflect meaningful gradients in population-level siren activity while ensuring sufficient observations within each category for stable estimation. At the national level, the incidence of dog bite diagnoses was compared across these categories to assess whether months characterized by exposure to more civil defense sirens were associated with higher rates of dog bite injuries. Analyses were additionally stratified by geographic region (North, Center, South) to evaluate whether regional increases in exposure to civil defense sirens were associated with a higher regional dog bite incidence during the corresponding months.

### Statistical analysis

Monthly counts of dog bite-related healthcare encounters were modeled using negative binomial regression to account for overdispersion in the count data (variance exceeding the mean), for which Poisson assumptions were not met. The dependent variable was the monthly number of dog bite incidents. The primary exposure was monthly siren activity, categorized a priori by level of exposure (0, < 500, or ≥ 500 sirens). Models included geographic region and were adjusted for seasonality and relevant demographic and socioeconomic covariates, including age group, sex, and socioeconomic status. Seasonality was modeled using a categorical variable for calendar month. A continuous time variable was included to account for secular trends over the study period. Because the unit of analysis was the region-month, individual-level covariates (age group, sex, and socioeconomic status) were incorporated as aggregated variables. For each region-month observation, these variables were summarized as proportions of the total number of dog bite cases and included as covariates in the regression models. Incidence rate ratios (IRRs) with 95% confidence intervals (CIs) were reported. Statistical analyses were performed using R (version 4.3.2), and statistical significance was defined as a two-sided α level of 0.05.

### Analytical considerations

The analysis reflects associations at the region-month level and does not capture immediate temporal relationships between individual siren events and dog bite occurrences.

Although the data are structured over time, formal time-series modeling approaches incorporating autocorrelation were not applied.

The statistical analysis code is available from the corresponding author upon reasonable request.

### Ethics approval

This study was based on the analysis of anonymized, retrospective administrative healthcare data and did not involve direct contact with human or animal subjects. The study was approved by the Institutional Review Board (Helsinki Committee) of Rabin Medical Center.

## Results

A total of 63,285 dog bite-related healthcare encounters were identified during the study period, of which 36,661 occurred during months with no sirens, 18,971 during months with low siren exposure (< 500 sirens), and 7,653 during months with high siren exposure (≥ 500 sirens). The demographic, socioeconomic, geographic, seasonal, and clinical characteristics of the dog bite cases stratified by siren exposure status are summarized in Table 1.

**Table 1:** Patient, environmental, and dog-bite characteristics by level of siren exposure*

Variable	Overall *n* = 63,285	No sirens *n* = 36,661	Low exposure *n* = 18,971	High exposure *n* = 7,653
Age group (years)				
0–4	4,825 (7.6%)	2,667 (7.3%)	1,571 (8.3%)	587 (7.7%)
5–14	16,572 (26%)	9,523 (26%)	5,184 (27%)	1,865 (24%)
15–24	10,101 (16%)	5,919 (16%)	2,989 (16%)	1,193 (16%)
25–44	15,871 (25%)	9,290 (25%)	4,637 (24%)	1,944 (25%)
45–64	9,805 (15%)	5,716 (16%)	2,870 (15%)	1,219 (16%)
>65	6,111 (9.7%)	3,546 (9.7%)	1,720 (9.1%)	845 (11%)
Sex				
Female	25,164 (40%)	14,357 (39%)	7,573 (40%)	3,234 (42%)
Male	38,121 (60%)	22,304 (61%)	11,398 (60%)	4,419 (58%)
Socioeconomic status^†^				
Low	12,325 (19%)	7,236 (20%)	3,773 (20%)	1,316 (17%)
Medium	34,131 (54%)	19,812 (54%)	10,082 (53%)	4,237 (55%)
High	12,569 (20%)	7,510 (20%)	3,453 (18%)	1,606 (21%)
No data	4,260 (6.7%)	2,103 (5.7%)	1,663 (8.8%)	494 (6.5%)
Region				
South	9,141 (14%)	1,641 (4.5%)	6,623 (35%)	877 (11%)
Center	33,150 (52%)	20,375 (56%)	8,124 (43%)	4,651 (61%)
North	20,994 (33%)	14,645 (40%)	4,224 (22%)	2,125 (28%)
Season of injury				
Spring	17,012 (27%)	9,625 (26%)	5,362 (28%)	2,025 (26%)
Summer	17,048 (27%)	9,632 (26%)	5,241 (28%)	2,175 (28%)
Autumn	15,633 (25%)	8,625 (24%)	4,245 (22%)	2,763 (36%)
Winter	13,592 (21%)	8,779 (24%)	4,123 (22%)	690 (9.0%)
Hospitalization within 7 days	452 (0.7%)	262 (0.7%)	131 (0.7%)	59 (0.8%)
Surgical intervention within 30 days	11,389 (18%)	6,591 (18%)	3,382 (18%)	1,416 (19%)

Values are presented as n (%)

* Siren exposure was defined by the number of monthly regional civil defense sirens: none (0), low (< 500), and high (≥ 500)

^†^Socioeconomic status was classified according to the national socioeconomic index

Months with low siren exposure (< 500 sirens) were associated, on average, with a 15% increase in monthly dog bite incidence (IRR 1.15, 95% CI 1.13–1.18), and months with high exposure (≥ 500 sirens) with a 33% increase (IRR 1.33, 95% CI 1.28–1.37), after adjustment for geographic region, seasonality, age group, sex, and socioeconomic status (Table 2).

**Fig. 1:**
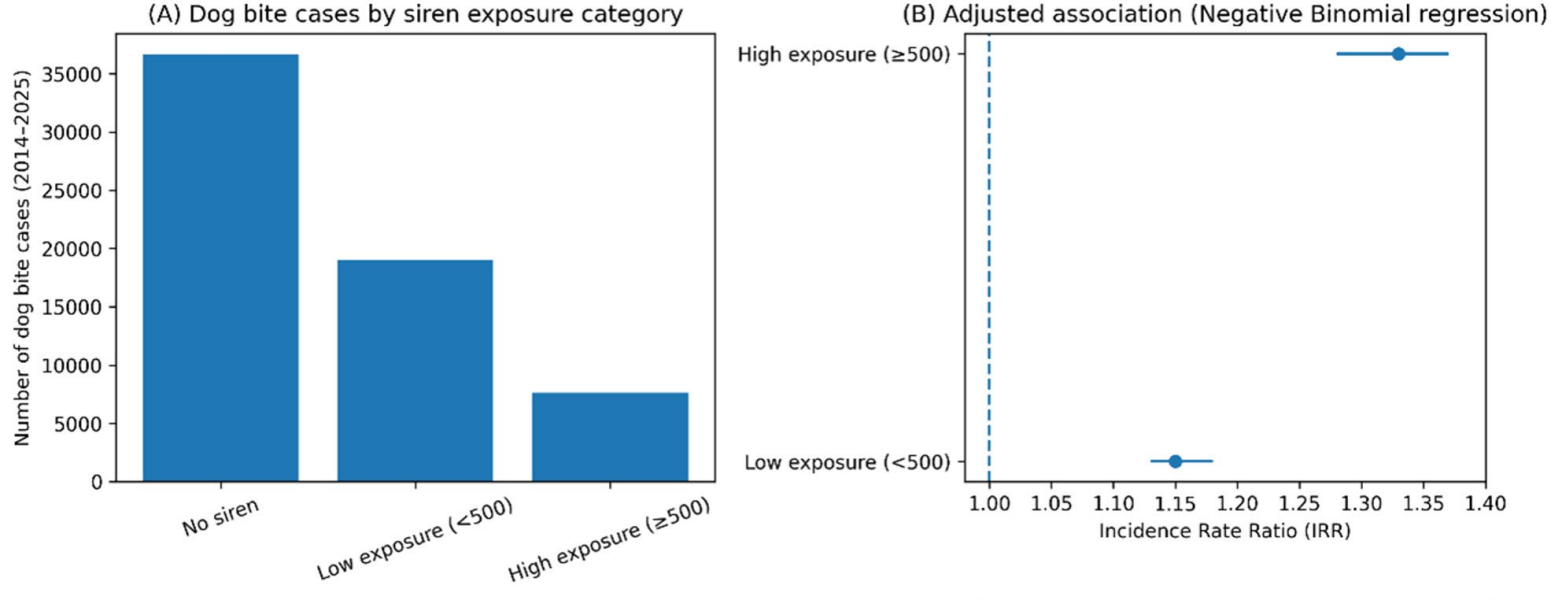
Dog bite-related healthcare encounters by siren exposure category in Israel (2014–2025). (**A**) Total number of dog bite-related healthcare encounters aggregated across the study period, stratified by monthly civil defense siren exposure: no sirens, low exposure (< 500 sirens per month), and high exposure (≥ 500 sirens per month). (**B**) Adjusted incidence rate ratios (IRRs) with 95% confidence intervals (CIs) for dog bite incidence by siren exposure category, estimated using multivariable negative binomial regression models. Months with no sirens served as the reference category (IRR = 1). Models were adjusted for geographic region, seasonality, age group, sex, and socioeconomic status

**Fig. 2:**
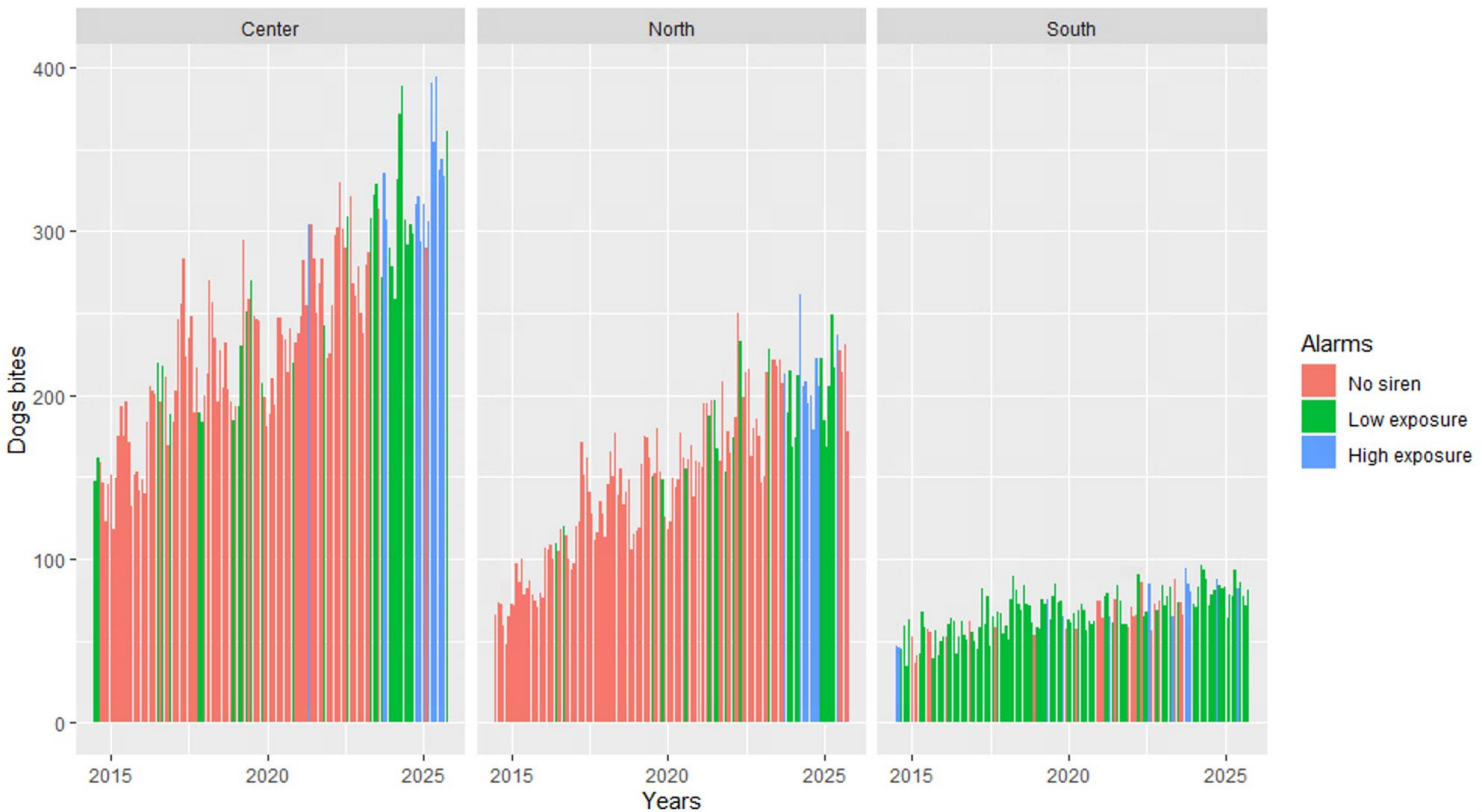
Monthly dog bite-related healthcare encounters by geographic region and siren exposure category in Israel (2014–2025). Bars represent monthly counts of dog bite-related healthcare encounters stratified by geographic region (Center, North, South) and level of civil defense siren exposure: no sirens, low exposure (< 500 sirens per month), and high exposure (≥ 500 sirens per month). This figure illustrates temporal and regional variation in dog bite incidence in relation to escalation-related environmental stress

The temporal patterns (Fig. 1) demonstrate a graded increase in dog bite incidence with higher levels of siren exposure, while regional stratification (Fig. 2) shows higher absolute counts in the Central region across all exposure categories, reflecting underlying population distribution.

**Table 2:** Association between siren exposure level and monthly dog bite incidence

Variable	IRR	CI 95%	*P*-value
Siren exposure category			
No siren	Reference	-	-
Low exposure (< 500 sirens)	1.15	1.13–1.18	< 0.001
High exposure (≥ 500 sirens)	1.33	1.28–1.37	< 0.001

Incidence rate ratios (IRRs) were estimated using negative binomial regression models adjusted for geographic region, seasonality, age group, sex, and socioeconomic status

IRR, incidence rate ratio; CI, confidence interval

Regarding secondary outcomes, 452 (0.7%) encounters were followed by hospitalization within 7 days and 11,389 (18.0%) by surgical intervention within 30 days (Table 1), indicating a substantial burden of operative management. In adjusted models, high siren exposure was associated with increased odds of surgical intervention within 30 days (OR 1.09, 95% CI 1.02–1.16; *P* = 0.013), whereas no statistically significant association was observed for hospitalization within 7 days (Table 3).

**Table 3:** Association between siren exposure level and injury severity among dog bite cases

Outcome	Siren exposure category	Adjusted OR	CI 95%	*P*-value
Surgical intervention within 30 days	No siren	Reference	-	-
	Low exposure (< 500 sirens)	1.04	0.99–1.10	0.11
	High exposure (*≥* 500 sirens)	1.09	1.02–1.16	0.013
Hospitalization within 7 days	No siren	Reference	-	-
	Low exposure (< 500 sirens)	1.06	0.85–1.33	0.60
	High exposure (*≥* 500 sirens)	1.09	0.81–1.44	0.60

Odds ratios (ORs) were estimated using multivariable logistic regression models adjusted for geographic region, seasonality, age group, sex, and socioeconomic status

OR, odds ratio; CI, confidence interval

## Discussion

In this nationwide retrospective study (2014–2025), we evaluated whether armed conflict-related environmental stressors, operationalized using civil defense siren activity, were associated with population-level variations in dog bite-related healthcare encounters. We identified a statistically significant graded association between the level of siren exposure and monthly dog bite incidence. This graded pattern is consistent with a dose-response relationship, suggesting that increasing levels of environmental stress during periods of escalation may contribute to higher rates of dog bite injuries at the population level. Importantly, this study provides, to our knowledge, the first national-level evidence linking armed conflict-related environmental stressors to increased dog bite incidence.

A key strength of this work is the use of siren alert data as a quantitative exposure measure. Civil defense sirens provide an objective, time-stamped, region-specific, and publicly documented indicator of environmental stress related to conflict escalation. Siren activity captures not only acute acoustic exposure but also broader contextual disruption during escalation periods, including shelter confinement, altered routines, and heightened mental strain, as described in studies of human-animal interactions and companion animal behavior during periods of environmental stress and routine disruption [11, 12, 14, 15], allowing for the assessment of population-level injury risk within a natural experiment framework. Notably, the graded siren exposure categorization offers a reproducible approach that extends beyond binary definitions of conflict versus non-conflict periods and enables evaluation of dose-response patterns at the population level.

The observed association is biologically and behaviorally plausible [8–10]. High-intensity auditory stimuli have been associated with anxiety-related behavioral dysregulation in dogs, and companion animals are also known to be sensitive to changes in household stress and routine [5–7]. However, the present study was not designed to isolate a single mechanistic pathway. Rather, it adopts an ecological injury-risk perspective in which escalation-related stressors are expected to act simultaneously across multiple domains, including environmental context, human behavior and supervision patterns, and stress-related responses in dogs, resulting in a net population-level change in dog bite risk. Importantly, these pathways are not mutually exclusive and likely co-occur during periods of heightened threat exposure.

Additionally, we assessed clinically meaningful severity endpoints reflecting healthcare utilization and system burden rather than behavior alone. Hospitalization within 7 days was uncommon and not significantly associated with siren exposure category. However, high-exposure months were associated with increased odds of surgical intervention within 30 days (OR 1.09, 95% CI 1.02–1.16). Although the effect size was small, it may be operationally relevant given the high baseline volume of dog bite encounters. The relatively high rate of surgical intervention (18%) underscores the clinical significance of dog bite injuries in this population and highlights the potential burden on surgical services. Even modest increases in operative management may translate into a measurable surgical workload during periods when healthcare systems are simultaneously managing many escalation-related demands.

Regional differences were observed in the adjusted models, with lower dog bite incidence in the North and South of the country compared with the Central region. The descriptive patterns shown in Fig. 2 similarly indicate higher monthly counts of dog bite-related encounters in the Central region over the study period. However, the interpretation of geographic differences in dog bite incidence in Israel requires careful consideration of population distribution and displacement dynamics during escalation periods. The Central region includes a substantial proportion of the national population and has a higher population density, which may influence baseline exposure patterns and healthcare utilization. In addition, in Israel, escalation periods have historically involved a disproportionate siren burden in the South and, more recently, in the North. They may also involve substantial internal displacement and temporary relocation of residents from conflict-adjacent areas to central regions, altering both the geographic denominators at risk and the location of healthcare utilization. Thus, dog bite encounters may be redistributed toward the Central region irrespective of where exposure occurred. Consequently, region-level comparisons of dog bite incidence should be interpreted as reflecting both underlying demographic structure and time-varying population movement rather than stable geographic differences in risk. Future research should further explore region-specific dynamics using more granular population denominators and mobility data to better disentangle true geographic variation in risk from shifts in population distribution during periods of escalation.

These findings have actionable public health and injury prevention implications. Preparedness during escalation periods, particularly in high-exposure areas, may benefit from incorporating dog bite injury mitigation into emergency department and surgical resource planning. Public guidance issued during siren events could include practical dog-handling recommendations, emphasizing child supervision, minimization of high-risk interactions in confined environments such as shelters, and use of restraints when feasible. Dog bite prevention may therefore represent a previously underrecognized component of civilian injury prevention during armed conflict. Taken together, these findings likely reflect a complex interplay between environmental stress exposure, human behavior, and contextual factors that vary across populations and settings. Although the present analysis focused on population-level associations, the observed patterns may differ across demographic and geographic subgroups and over time, suggesting additional layers of heterogeneity that warrant further investigation.

Several limitations merit consideration. This study relied on diagnostic coding and captured dog bite events resulting in healthcare utilization. Under-ascertainment of mild injuries is likely in routine periods and may be more pronounced during escalation periods when individuals may avoid seeking care for minor wounds. Such misclassification would be expected to be largely nondifferential and to bias effect estimates toward the null, suggesting that the observed associations may be conservative. In addition, data on dog breed, household structure, and individual-level behavioral factors were unavailable, limiting mechanistic inference. Information on the ownership status of the biting dog was not available. Although post-exposure rabies prophylaxis could potentially serve as a proxy for this distinction, these data were not accessible for linkage in the present study. Additional environmental acoustic stressors, such as explosions or gunfire, were not directly measured, as standardized and time-resolved data on these events were not available for linkage. Civil defense sirens, which are systematically recorded, were therefore used as a practical proxy and likely capture broader escalation-related acoustic exposure at the population level. The categorization of siren exposure was based on predefined thresholds in the available dataset and not on externally validated cut-offs, which may limit the precision of exposure classification. The ecological design does not permit causal attribution at the individual level, and residual confounding may persist despite adjustment for demographics, seasonality, and region. Potential effect modification by demographic and geographic factors was not explored and may provide additional insight into vulnerable populations. Environmental factors such as temperature and precipitation were not included in the models and may contribute to seasonal variation in dog bite incidence and severity. Alternative modeling approaches, including continuous parameterization of siren counts and non-linear modeling, were not explored and may provide additional insights into the dose-response relationship. More advanced analytical approaches, such as case-crossover designs or distributed non-linear lag models, were not applied in the present study but may provide additional insight into temporal dynamics and potential lagged effects in future analyses.

In summary, using a nationwide dataset spanning more than a decade, we demonstrate a graded association between escalation-related environmental stress, quantified through civil defense siren burden, and population-level dog bite incidence. By leveraging a quantitative exposure measure rather than a binary conflict definition, this study strengthens evidence that armed conflict-related environmental stressors can modify civilian injury risk. These findings support integrating dog bite prevention and healthcare preparedness into civilian injury mitigation strategies during armed conflict.

## Data Availability

The datasets analyzed during the current study are based on anonymized administrative healthcare data and are subject to institutional data access regulations. Therefore, the data are not publicly available but may be accessed upon reasonable request and with appropriate institutional approvals.

## Abbreviation

CHS: Clalit Health Services
